# Editorial for This Special Issue “Synaptic Transmission: From Molecular to Neural Network Levels”

**DOI:** 10.3390/biomedicines12010145

**Published:** 2024-01-10

**Authors:** Lisa Mapelli, Simona Tritto

**Affiliations:** Department of Brain and Behavioral Sciences, University of Pavia, 27100 Pavia, Italy

We invited contributions reporting new evidence of synaptic mechanisms and their network-level impacts for this Special Issue. The six research articles published in this collection, from 33 authors, cover many aspects of synaptic function and plasticity, from single-cell mechanisms to neuronal network properties. These papers elucidate the physiological and pathological conditions underlying synaptic transmission, giving new perspectives for future applications and therapies. The main results are summarized below.

Oscillation and resonance are critical neural network properties that characterize the activity of several brain regions, from the neocortex to the cerebellum [1,2]. In particular, the presence of recurrent networks and reverberating loops allows for activity coordination among different regions and the generation of further sustained oscillations. The cerebellar network is known to operate at different time scales, and researchers have extensively investigated dynamic properties such as oscillation and resonance, mainly at the cortical input layer and deep cerebellar nuclei. In this Special Issue, Bauer and colleagues investigate whether Purkinje cells, which represent the unique output of the cerebellar cortex and are the most studied cerebellar neurons for their computational power, show a preference for specific frequency bands in their motor activity control. This study focuses on controlling the olivocerebellar circuit for whisker movements in awake mice. Purkinje cells receive their inputs from granule cell axons, the parallel fibers, controlling simple spikes production, and from inferior olive neuron axons, the climbing fibers, eliciting the so-called complex spikes. By combining the extracellular single-unit recordings and optogenetic manipulations of Purkinje cell activity, this study revealed that Purkinje cell activation at 8 Hz induces the largest whisker deflection, showing that these neurons work preferentially in the theta band. This observation agrees with the evidence on the granular layer and deep cerebellar nuclei, which show oscillations and resonance in the theta band [3,4]. Moreover, Bauer and colleagues provide evidence that the simple spike pathway activates reverberating loops, contrary to climbing fiber activation, which is, nevertheless, effective in inducing movements. The authors hypothesize this difference might be due to a lack of deep cerebellar nuclei inhibition preceding the disinhibition obtained via the climbing fiber activation of Purkinje cells. The need for a first inhibitory phase of deep cerebellar nuclei neurons to demonstrate an oscillatory behavior is in line with previous results where activity-dependent changes in nuclei neuron firing depended on the ability of these neurons to oscillate in the theta band, a condition observed only in those neurons showing inhibition as the first response phase [4].

In a similar effort to elucidate the frequency dependence of cerebellar activity, Monteverdi and colleagues dissected the dynamic properties of input processing in the cerebellar cortex by using high-density multi-electrode array recordings in acute slices. They evaluated the cortical circuit short-term dynamics in the granular and Purkinje cell layers induced by stimulating mossy fibers, which represent the other cerebellar input besides the climbing fibers. This study shows that the cerebellar cortical network is characterized by a notable frequency dependence in all its layers with differences in the sagittal and coronal orientations of the circuit, highlighting the anisotropy of signal processing. Moreover, these results show that the circuit can efficiently process and transmit low-frequency inputs. This was a previously underestimated property of this circuit, considering the ability of granule cells to emit spikes at fast rates with sub-millisecond precision [2], which drove most cerebellar research towards faster activity rates. This evidence aligns with the oscillatory and resonant properties of the cerebellar network, the findings reported by Bauer and colleagues, and the recently revalued cerebellar role in the supra-second domain [5]. Monteverdi and colleagues conclude that cerebellar dynamic processing is governed by a complex set of spatiotemporal filters characterized by a notable frequency dependence and an anisotropy that is likely to reflect the anatomical organization of local inhibition.

In the paper by Mapelli et al., another aspect of synaptic plasticity in the cerebellar granular layer was considered. By combining patch-clamp intracellular recordings and mathematical modeling, the authors show that long-term plasticity at the cerebellar input stage can alter the gain bandwidth of mossy fiber burst transmission. It is known that the mossy fiber–granule cell relay can differentially undergo LTP or LTD depending on the granule cell depolarization attained during the induction [6]. The novelty of the paper by Mapelli et al. lies in the finding that long-term plasticity can tune the gain and timing of transmission along channels at the cerebellum input stage. The spatiotemporal reconfiguration of the mossy fiber–granule cell input–output transmission is a gain-controlled process dependent on long-term plasticity. Moreover, the different effects of long-term depression and potentiation strongly affect the time and frequency domains of single-transmission channels. These results confirm the hypothesis that the cerebellum can behave as an adaptive spatiotemporal filter. This spatiotemporal organization in the center–surround characteristics structures reflects a fine-tuning of the excitatory/inhibitory balance between the center (with a higher E/I ratio) and the surround (with a lower E/I ratio) due to the differential activation of glutamate and GABA receptors. The voltage-dependent NMDA receptors are regulated accordingly, enhancing burst transmission in response to their long integration time constant and inducing synaptic plasticity thanks to their calcium permeability. In the center, larger depolarizations would enhance NMDA channel voltage-dependent unblocking, improving synaptic integration at low frequencies and simultaneously raising the calcium influx, facilitating LTP induction [7]. The computational model confirmed that the changes in the GABAergic inhibition-mimicking plastic mechanisms were required to attenuate the marked changes in gain curves occurring when only excitatory plasticity was present. GABAergic plasticity acts as a homeostatic regulatory mechanism avoiding the saturation of synaptic weights and maintaining a dynamic range of activity in both time and frequency domains. The authors conclude that the cerebellar granular layer activity can be reorganized by LTP and LTD tuning the firing composition in terms of spike delay and fine-tuning the number and frequency of spikes. Moreover, LTP and LTD can extend the transmission bandwidth toward lower frequencies.

Parkinson’s disease (PD) is a neurodegenerative condition traditionally related to the classic motor phenotype that also shows clear cognitive symptoms [8]. Though the clinical manifestations show a high degree of heterogeneity, most genes and proteins implicated in its pathogenesis are directly or indirectly related to the synaptic function, among which one of the most renowned examples is alpha-synuclein. For this reason, PD is considered a synaptopathy [9]. In this Special Issue, Oey and colleagues applied quantitative proteomics and RNA interference techniques in rat primary cortical neuron cultures to describe the effects of knocking down the expression of PHF8 in synaptically activated neurons. PHF8 is a histone demethylase involved in PD and X-linked intellectual disability. This study reports that PHF8 silencing in activated neurons results in the downregulation of several proteins involved in synaptic structure, function, and plasticity, both at the pre- and postsynaptic site, including alpha-synuclein, calcium-calmodulin-dependent kinase II alpha (CaMKIIa), complexin 1, and other proteins found involved in PD. In particular, alpha-synuclein has been causally related to PD for its role in dopaminergic transmission [9], and CaMKIIa is known to play a critical role in synaptic transmission and plasticity, including dopaminergic connections [10]. Interestingly, these results suggest that large clusters of functionally related proteins are affected by the downregulation of PHF8. This evidence deserves further investigation in light of its potential impact on novel approaches to disease-modifying therapies [11].

Pimozide is an antipsychotic drug known to inhibit synaptic transmission in the central nervous system, acting as a dopamine D2 receptor antagonist [12], and is, therefore, used for schizophrenia and other neurological disorder treatments [13]. Pimozide is also known for its side effects, including akinesia, tremor, and altered vestibular function. In fact, blocking D2 receptors inhibits synaptic transmission, while cell depolarization produced by blocking K^+^ channels may increase synaptic transmission. Giunta and co-authors investigated the effects of pimozide on the ionic currents of voltage-gated K^+^ channels recorded from chicken embryo vestibular type-II hair cells using patch-clamp whole-cell recordings in the ex vivo slices of semicircular canals obtained at different developmental stages. The authors demonstrated that pimozide increased the amplitude of *I_KV_* elicited at negative potentials in vestibular type-II hair cells. This effect was still present at a lower concentration, while the already-known blocking effect disappeared (3 µM vs. 0.3 µM). Moreover, pimozide significantly hyperpolarized the resting membrane potential of type-II hair cells. This would close voltage-gated Ca^2+^ channels at the basolateral side, which are involved in the glutamate exocytosis at the afferent nerve terminal of vestibular primary sensory neurons. Altogether, these results suggest that the hyperpolarization of vestibular hair cells is related to the decrease in glutamate release due to the pimozide effect. It is known that vestibular dysfunctions are very common in adults [14], and vertigo is one of the main symptoms due to abnormal signaling from vestibular hair cells. Usually, the treatment in the case of severe vestibular symptoms, such as vertigo, is the chemical or surgical ablation of the vestibular nerve, but this can lead to permanent hearing loss. Therefore, pimozide, which has no hearing loss side effects, could be a good candidate for vestibular disorder treatment. Moreover, the authors demonstrated that pimozide acts as a K^+^ channel opener and could be a promising drug treatment also for other neurological disorders.

During the last decade, the consumption of sugar-sweetened beverages has dramatically increased, and it has been demonstrated that it can impair brain function [15]. Brain-derived neurotrophic factor (BDNF) is a key molecule involved in plastic changes related to learning and memory, and its expression is highly regulated [16]. In this Special Issue, Coirini et al. demonstrated that juvenile rats exposed to high-sugar beverages show reduced mRNA expressions of BDNF and hippocampus inflammation. Novel object recognition (NOR) tests were performed in juvenile and adult rats exposed to 10% sucrose beverages. The study by Coirini et al. demonstrates impairment in NOR only in juvenile rats, excluding attention deficits, as the time spent in the exploring phase was comparable in the two groups. Moreover, while the authors previously reported long-term changes in fear memory conditions and anxiety behavior in juvenile rats exposed to sucrose [17], the behavioral conditions were optimized in the present research such that the animals showed no anxiety. Therefore, the cognitive defects observed were due to learning and memory impairment and not stress interference. The authors also showed that the BDNF precursor (proBDNF) decreased in juvenile rats exposed to sucrose, while it increased in adults; the decreased proBDNF expression was observed in the hippocampus correlated to decreases in the recognition memory. The authors also found an increased expression of the receptor for advanced glycation end-products (RAGE) in adults but without a correlation with deficit cognition. In conclusion, lower levels of proBDNF in the hippocampus may be the cause of the cognitive deficits observed in adult animals exposed to sucrose during youth.
